# The aryl hydrocarbon receptor is expressed in non-sensory cells in the cochlea and regulates hair cell survival

**DOI:** 10.3389/fnmol.2026.1943479

**Published:** 2026-09-18

**Authors:** Sujata Pandey, Brandon C. Cox

**Affiliations:** 1 Department of Pharmacology, Southern Illinois University School of Medicine, Springfield, IL, United States; 2 Department of Otolaryngology, Southern Illinois University School of Medicine, Springfield, IL, United States

**Keywords:** aminoglycoside antibiotics, aryl hydrocarbon receptor, ATP, hair cells, neomycin, noise exposure, supporting cells

## Abstract

Damage or loss of hair cells, the sensory cells for hearing, is caused by ototoxic drugs or acoustic trauma, and results in permanent hearing loss because the adult cochlea lacks regenerative capacity. Therefore, identifying pathways that regulate hair cell survival is critical. Here we investigate a novel pathway, aryl hydrocarbon receptor (AhR) signaling in the cochlea. We demonstrate for the first time, the expression pattern of AhR in the cochlea under physiological conditions and after exposure to neomycin or noise. Under physiological conditions, AhR was strongly expressed in glia surrounding spiral ganglion neurons with low expression in the organ of Corti. Following neomycin treatment or noise exposure, AhR expression increased in supporting cells suggesting a dynamic response to hair cell injury. This hair cell to supporting cell communication is mediated in part by purinergic signaling, since treatment with ATP mimicked the effect of neomycin, and treatment with the purinergic antagonist suramin eliminated AhR upregulation. In addition, deletion of *AhR* from the organ of Corti significantly increased hair cell loss after neomycin treatment compared to controls with normal *AhR* levels. Our findings establish AhR as a cellular biosensor in supporting cells which is upregulated during hair cell injury to regulate the survival of hair cells.

## 1 Introduction

Hearing loss affects nearly 1.6 billion people worldwide and continues to rise, driven by factors such as ototoxic drugs, noise exposure, and aging (Haile et al., 2021; Prasad et al., 2024). In mammals, permanent hearing impairment largely results from the loss of cochlear hair cells, which lack regenerative capacity in the adult cochlea (Bohne et al., 1976; Hawkins et al., 1976; Oesterle et al., 2008). Because hair cell loss is permanent, there is a critical need to identify endogenous signaling pathways that can sense cellular stress and promote protective or homeostatic responses within the cochlea.

The aryl hydrocarbon receptor (AhR) is a ligand-dependent transcription factor in the basic helix–loop–helix PER-ARNT-SIM (bHLH-PAS) family that functions as a cellular biosensor (Poland et al., 1976; Hoffman et al., 1991; Burbach et al., 1992; Reyes et al., 1992; Pongratz et al., 1998; Nebert, 2017; Schulte et al., 2017). AhR is activated by various ligands such as environmental toxins, xenobiotics and endogenous metabolites that result in different outcomes (Nguyen and Bradfield, 2008; Mulero-Navarro and Fernandez-Salguero, 2016). The biological outcome of AhR signaling is determined primarily by the specific structural and chemical properties of the bound ligand inducing unique conformational changes in the receptor (Denison and Faber, 2017; Nebert, 2017; Diao et al., 2025; Coumoul et al., 2026). Some AhR ligands act as environmental toxins such as 2,3,7,8-Tetrachlorodibenzo-p-dioxin (TCDD) to damage cells, while those derived from tryptophan metabolism, have a protective function (Nebert et al., 1999; van den Berg et al., 2013; Dietrich, 2016; Hou et al., 2023). Upon ligand binding, AhR translocates to the nucleus and regulates gene expression to control pathways involved in detoxification, inflammation and cellular homeostasis (Durrin et al., 1987; Hankinson, 1995; Gu et al., 2000; Wright et al., 2017; Marinelli, 2020; Riaz et al., 2022). These properties make AhR a potential candidate for mediating stress-response signals in tissues exposed to environmental challenges and stressors. However, despite its well-characterized role as a cellular biosensor that can provide anti-oxidative and anti-inflammatory effects in other cell types, AhR signaling has not been well studied in the cochlea.

In the central nervous system, AhR is expressed in astrocytes, microglia, oligodendrocytes and neurons where it regulates neuronal homeostasis, modulates inflammatory responses, and supports adaptation to injury resulting in repair or protection of neurons (Petersen et al., 2000; Filbrandt et al., 2004a; Williamson, 2004; Latchney et al., 2013a; Shackleford et al., 2018; Linnerbauer et al., 2020; Rothhammer et al., 2021a; Sun et al., 2026a). Deletion of *Ahr* increases the production of pro-inflammatory cytokines *in vitro* and accentuates the development of an inflammatory astrocytic phenotype *in vivo*, in response to two different inflammatory signals, lipopolysaccharides (LPS) (Ojo and Tischkau, 2025) and amyloid-β (Ojo et al., 2025). In addition, activation of AhR by the endogenous ligand, FICZ, can prevent neuronal cell death, whereas AhR inhibition promotes LPS-induced neurotoxicity (Gan and Lee, 2025). Notably, astrocytes and microglia share functional similarities with cochlear supporting cells (Bermingham-McDonogh and Reh, 2011; Monzack and Cunningham, 2013; Wan et al., 2013), suggesting a conserved role for AhR in coordinating responses to stress. This parallel provides a strong rationale for investigating AhR in the cochlea, particularly in the context of cell–cell communication.

The organ of Corti, the auditory organ located within the cochlea, relies on tightly coordinated interactions between sensory hair cells and surrounding supporting cells to maintain function and to respond to injury. Supporting cells play an active role in preserving cochlear homeostasis by regulating ionic balance, clearing damaged cells through phagocytosis, releasing trophic factors, taking up neurotransmitters and participating in repair processes in the organ of Corti (Glowatzki et al., 2006; Zhao et al., 2008; Wan et al., 2013; Hayashi et al., 2020; Ding et al., 2025). Transcriptomic studies have identified AhR expression in supporting cells under normal conditions (Scheffer et al., 2015; Perl et al., 2018) and upregulation of *AhR* following hair cell damage in regenerative models such as the chicken basilar papilla (Hawkins et al., 2007). These findings suggest that AhR may function as a sensor of cellular stress, enabling supporting cells to modulate the local microenvironment and influence hair cell survival. Based on this and AhR’s role in astrocytes, we hypothesized that AhR promotes hair cell survival by mediating communication between supporting cells and hair cells during injury. In this study, we investigated the expression pattern of AhR in the normal and damaged cochlea and the mechanism of intercellular communication between injured hair cells and supporting cells mediating changes in AhR expression. We also examined the endogenous role of AhR in modulating hair cell survival following neomycin induced injury.

## 2 Materials and methods

### 2.1 Mouse model

*AhR^tdTomato^* reporter mouse line (stock #38111) (Diny et al., 2022), *Sox2^CreERT2^* (stock #17593) (Arnold et al., 2011) and *AhR^loxP/loxP^* (stock #6203) (Walisser et al., 2005) were purchased from the Jackson Laboratory (Bar Harbor, ME). *Sox2^CreERT2^:AhR^loxP/loxP^* mice were injected with tamoxifen (3 mg/40 g) body weight (BW), interperitoneally (IP) (Sigma-Aldrich, St. Louis, MO) at postnatal day (P)0. All animal work was performed in accordance with approved animal protocols from the Institutional Animal Care and Use Committee at Southern Illinois University School of Medicine.

### 2.2 Noise exposure

Prior to the experiment, the acoustic environment was calibrated using a microphone (Bruel & Kjaer, Type 4,191, No 2366520) and Bruel & Kjaer-PULSE Labshop Version 13.5 software. Sound pressure levels (SPL) were measured across the enclosure, measuring all four corners and the central area of the cage to confirm a consistent sound within the target range of 105 ± 5 dB SPL. Mice were exposed to noise (8–16 kHz at 105 ± 5 dB SPL) for 1 h between 10 a.m. to 1 p.m. Two mice were placed in a custom-designed cage, divided equally by a mesh wire partition to ensure individual containment while maintaining uniform sound exposure. No bedding or enrichment was present during the exposure.

### 2.3 Cochlear dissection and tissue preparation

Mice were anesthetized using isoflurane, decapitated and the temporal bones were removed and fixed in 4% paraformaldehyde (PFA) prepared from 16% PFA (Polysciences, Warrington, PA) in 10 mM phosphate-buffered saline (PBS) (Sigma-Aldrich) for 2 h at room temperature. After fixation, the temporal bones were decalcified in 120 mM EDTA (Sigma-Aldrich) for 48 h, transferred to 10 mM PBS and stored at 4 °C until use. For whole mount cochlear microdissections, the fixed cochlea were micro-dissected to obtain three equal cochlear turns (apex, middle and base) and stored in PBS at 4 °C (Montgomery and Cox, 2016).

For mid-modiolar sections, tissues were placed in a sucrose solution gradient from 20% sucrose overnight to 10% sucrose for 30 min in 10 mM PBS at 4 °C. The cochlea was embedded in optimal cutting temperature (OCT) media (Fisher Health Care, Woodbury, NY) and sectioned at 10 μm thickness using a cryostat (Epredia Microm HM525, Fisher Scientific, Hampton, NH). Sections were mounted on charged glass slides.

### 2.4 Cochlear explant culture

Cochleae from P2/P3 pups were micro-dissected to prepare cochlear explants. The explant dissection removes the lateral wall (including the stria vascularis and spiral ligament) as well as most of the spiral ganglion. Therefore, explants consist primarily of the sensory epithelium (organ of Corti) and some spiral ganglion neurites. The explants were placed on Millicell cell culture inserts (Sigma-Aldrich) which were coated with Cell-Tak (Discovery Labware, Inc., Bedford, MA) and incubated overnight in DMEM media (Gibco #10564–011, Grand Island, NY) containing 10% fetal bovine serum (FBS) (Gibco #26140079), 1% N2 supplement (Gibco, #17502001), 0.1% penicillin (Fisher Scientific, #BP29555) and amphotericin B (Gibco, #15290018) at 37 °C. Explants were transferred to antibiotic-free media supplemented with 2% FBS and N2 supplement and treated with 600 μM neomycin (Sigma-Aldrich, #N1442) or 100 μM ATP (Fisher Scientific, Eugene, OR) for 24 h.

For *AhR^TdTomato^* pups, after 24 h of neomycin or ATP treatment, explants were fixed in 4% PFA for 30–45 min at room temperature, then processed for immunostaining to assess changes in Tomato expression. For *Sox2^CreER^:AhR^loxP/loxP^* pups, after 24 h of neomycin treatment, explants were washed with media four times and further incubated in media for 24 or 48 h before fixation.

### 2.5 ATP measurements

For the ATP assay, cochlear explants were created from *C57BL/6 J* mice at P2 and treated with neomycin for 24 h. Next, the cochlear sensory epithelium was lysed with ATP lysis buffer (Fisher Scientific, #16189) by vigorous mixing to induce cell lysis in 1.5 mL centrifuge tubes. The lysates were then added to the standard reaction solution prepared from an ATP determination kit (Fisher Scientific, #A22066), which utilizes the luciferase-luciferin reaction to detect and quantify ATP levels. The luminescent signal and ATP concentration was measured in a white opaque 96 well plate (Fisher Scientific, #15042) using a microplate reader (BioTek Synergy, Winooski, VT).

### 2.6 Real time qPCR

RNA was extracted from cochlear explants using RNeasy® MicroKit (Qiagen, #74004). Four explants were pooled to generate one sample for RNA extraction. Samples were reverse transcribed for cDNA synthesis using maxima First Strand cDNA synthesis kit (Fisher Scientific, #K1641) followed by quantitative polymerase chain reaction (qPCR) (Luminaris Color HiGreen MasterMix, Fisher Scientific, #K0391) using a CFX Connect Optics Module (Biorad, Hercules, CA). Primers for *AhR* were purchased from Integrated DNA Technologies (Coralville, IA); Forward Primer: 5’-TTCTTAGGCTCAGCGTCAGCT-3’and Reverse Primer: 5’-GCAAATCCTGCCAGTCTCTGA-3′. Samples were run in triplicate with *RPl19* (Forward Primer: 5’GGTCTGGTTGGATCCCAATG-3′ and Reverse Primer: 5’-CCCGGGAATGGACAGTCA-3′) used as the housekeeping gene. Expression levels were compared using the Pfaffl method (Pfaffl, 2001) which includes primer efficiency in the delta–delta Ct equation.

### 2.7 Immunofluorescent staining, confocal imaging and image analysis

The cochlear turns from whole mount dissection were immunostained as free-floating tissue as previously described (Montgomery and Cox, 2016). For the mid-modiolar sections, immunofluorescence was performed following a standard protocol (Coleman et al., 2009). Briefly, the tissue was blocked and permeabilized at room temperature for 1 h with a solution of 10% Normal Horse Serum (NHS) or Normal Goat Serum (NGS, Vector Labs, Burlingame, CA), 1% Bovine Serum Albumin (BSA, Fisher, Hampton, NH), and 1% Triton-X (Sigma, St. Louis, MO) in 10 mM PBS prior to the application of the primary antibodies. Primary antibodies (Table 1) were diluted in a solution of 10 mM PBS containing 5% NHS or NGS, 1% BSA, and 0.1% Triton-X and incubated overnight at room temperature or 37 °C. After overnight incubation with the primary antibodies, cochlear tissues were washed 3 times with 10 mM PBS for 10 min. Next, fluorophore-conjugated secondary antibodies were diluted 1:1000 in a solution of 5% NHS or NGS, 1% BSA, and 0.1% Triton-X in 10 mM PBS and incubated for 2 h in the dark. Following the secondary antibody incubation, samples were washed three times for 10 min each in 10 mM PBS followed by a 20-min incubation with Hoechst (1:500, Fisher Scientific) and mounted on slides using Prolong Gold (Fisher Scientific). Samples were imaged using a Zeiss LSM800 confocal microscope (Carl Zeiss, Oberkochen, Germany) and processed using Zen 3.0 lite software (Carl Zeiss). Quantification of cells was performed in 200 μm regions from each cochlear turn. Two images per turn were imaged and averaged for hair cell counts. For quantification, cochlear whole mounts or sections were imaged using a 40X oil immersion objective. Low magnification imaging of cochlear sections was performed at 20X or 10X.

**Table 1:** List of antibodies and experimental conditions for immunostaining.

Protein	Host	Dilution	Special procedures	Vendor/catalog number
Myosin VIIa	Rabbit	1:200	None	Proteus Biosciences, #25–6,790
RFP	Mouse	1:100	Signal enhancer	Rockland, #200–301-3795
Sox2	Goat	1:200	None	Santa Cruz, #sc-17320
Sox2	Mouse	1:200	IgG1-specific antibody	R&D Systems, #AF 2018
B-Tubulin III (Tuj1)	Mouse	1:500	IgG2a-specific antibody	Biolegend, #801201
S100A1	Rabbit	1:100	Signal enhancer	Abcam, #ab868
BLBP	Rabbit	1:200	Signal enhancer	Abcam, #ab32423

For some antibodies (Table 1), signal enhancer (Image-iT™ FX, Fisher Scientific) was applied for 30 min prior to the blocking and permealization step. For antibodies from the same host species, IgG-specific antibodies were used to target specific IgG subclasses and prevent cross-reactivity between primary antibodies.

### 2.8 Statistical analysis

Mice of both sexes were used in all studies and the sample size (*n*) represents the number of explants or mice analyzed per group. The two explants generated from the same mouse were used as independent samples. For the qPCR data, four explants were pooled to generate one sample. For the ATP assay, two explants were pooled to generate one sample. Controls were Cre-negative littermates that also received tamoxifen. All data are presented as mean ± standard error of the mean (SEM). Data was analyzed using a two-way ANOVA or a Student’s t-test using Graphpad Prism 9.0 (Graphpad Software Inc., La Jolla, CA). A *p* value ≤ 0.05 was considered significant.

## 3 Results

### 3.1 Constitutive expression of the AhR in the organ of Corti and spiral ganglion

To investigate expression of the AhR in the cochlea under normal, physiological conditions, we used a knockin reporter mouse line *Ahr^tdTomato^* where tdTomato expression is controlled by endogenous *AhR* regulatory elements. The *Ahr^tdTomato^* mouse line carries a CRISPR/Cas9 mediated insertion of a *P2A-tdTomato* sequence immediately upstream of the stop codon in the endogenous *Ahr* gene. Because the P2A peptide causes ribosomal skipping, the AhR protein and the tdTomato fluorophore are synthesized as one molecule from the same transcript with cleavage after protein translation, ensuring that tdTomato is a reliable proxy for AhR expression (Szymczak-Workman et al., 2012). Whole mount dissections and mid-modiolar sections were performed at key ages including postnatal day (P)0 and 8 weeks of age (sexual maturation).

Cochlear whole mount dissections showed robust and persistent expression of tdTomato in the region medial to the organ of Corti at P0 and 8 weeks of age, while the expression of tdTomato within the organ of Corti was very low (Figure 1A). We observed many tdTomato-positive and Sox2-positive Schwann cells encasing the spiral ganglion neuron (SGN) projections from Rosenthal’s canal to the region below inner hair cells at P0 (Figures 1B,C) (*n* = 5). At 8 weeks, there were fewer tdTomato-positive cells in this region creating a mosaic pattern (Figures 1D–F) (*n* = 6). We also observed tdTomato-positive cells in the region below and medial to the inner border and inner phalangeal cells at P0 and 8 weeks of age (Figures 1G–L). These cells displayed a distinct ovoid shape and location that is similar to the unique population of nestin-positive cells that have been previously described (Chow et al., 2015; Smeti et al., 2011). Mid-modiolar sections in the middle turn showed robust and persistent expression of tdTomato in the Sox2-positive satellite glial cells surrounding Tuj1-positive SGN cell bodies at P0 (Figures 2A–F) (*n* = 6). At 8 weeks, qualitative assessment showed a reduced number of tdTomato-positive satellite glia compared to P0 (Figures 2G–L) (*n* = 6). No differences in the tdTomato expression pattern were observed across cochlear turns.

**Figure 1:**
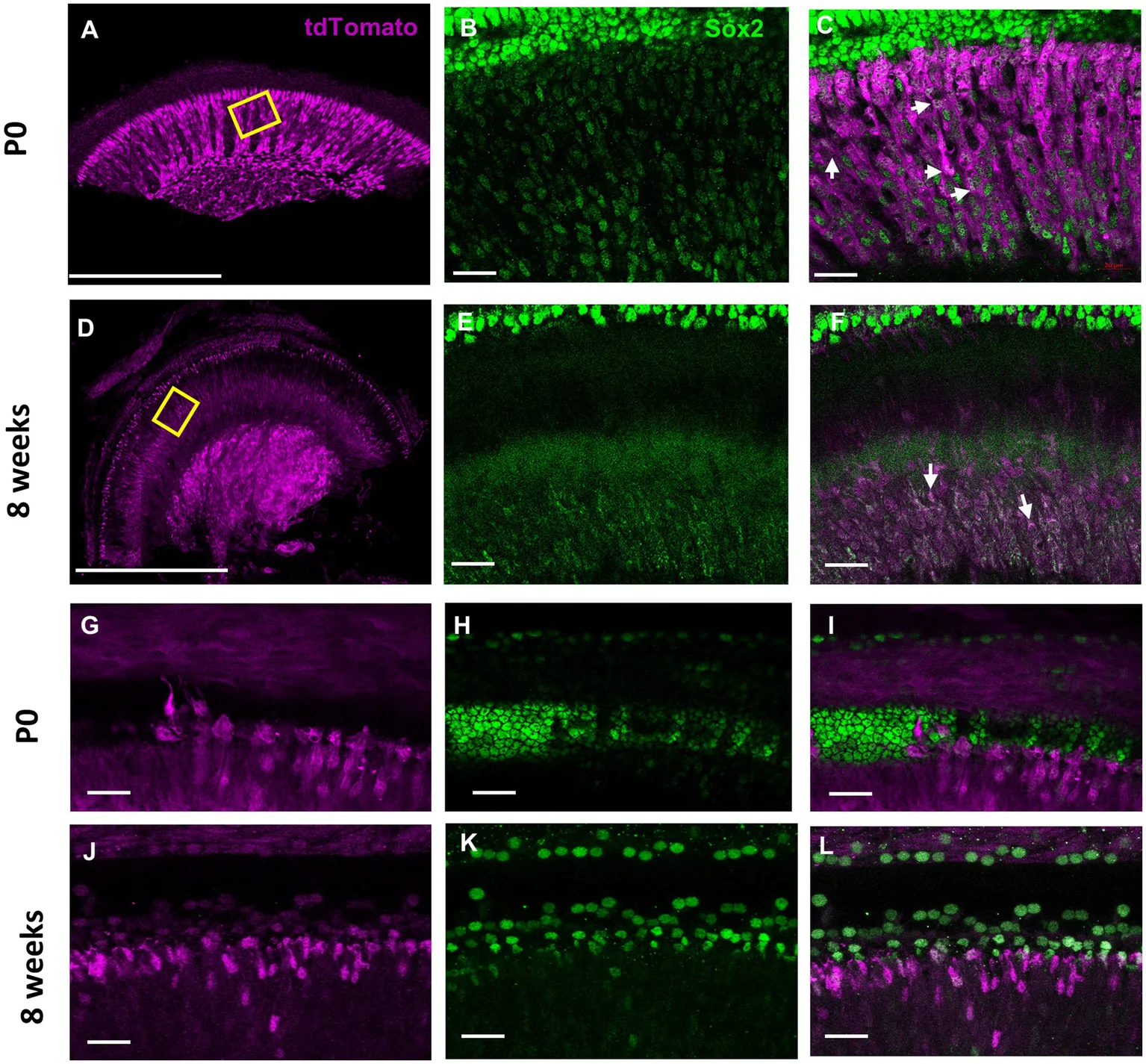
AhR is expressed in non-sensory cells under normal conditions at neonatal and adult ages. Confocal images showing tdTomato-positive cells (magenta) in the cochlea. (**A**) Whole mount of the middle turn at P0 (*n* = 5). **(B)** Sox2-positive cells Schwann cells (green) encasing the SGN projections from Rosenthal’s canal at P0. **(C)** tdTomato expression is present in the Sox2-positive Schwann cells (arrows) at P0. **(D)** Whole mount of the middle turn at 8 weeks of age. **(E)** Sox2-positive Schwann cells at 8 weeks of age (*n* = 6). **(F)** tdTomato expression is present in Sox2-positive Schwann cells (arrows) at 8 weeks of age. **(G–I)** tdTomato is expressed in the region medial to organ of Corti near Kolliker’s organ at P0. **(J–L)** At 8 weeks of age, tdTomato is expressed in non-sensory cells in the region below and medial to the inner phalangeal cell layer. Scale bar: 200 μm in **(A,D)**; 20 μm in **(B–C** and **E–H)**.

**Figure 2:**
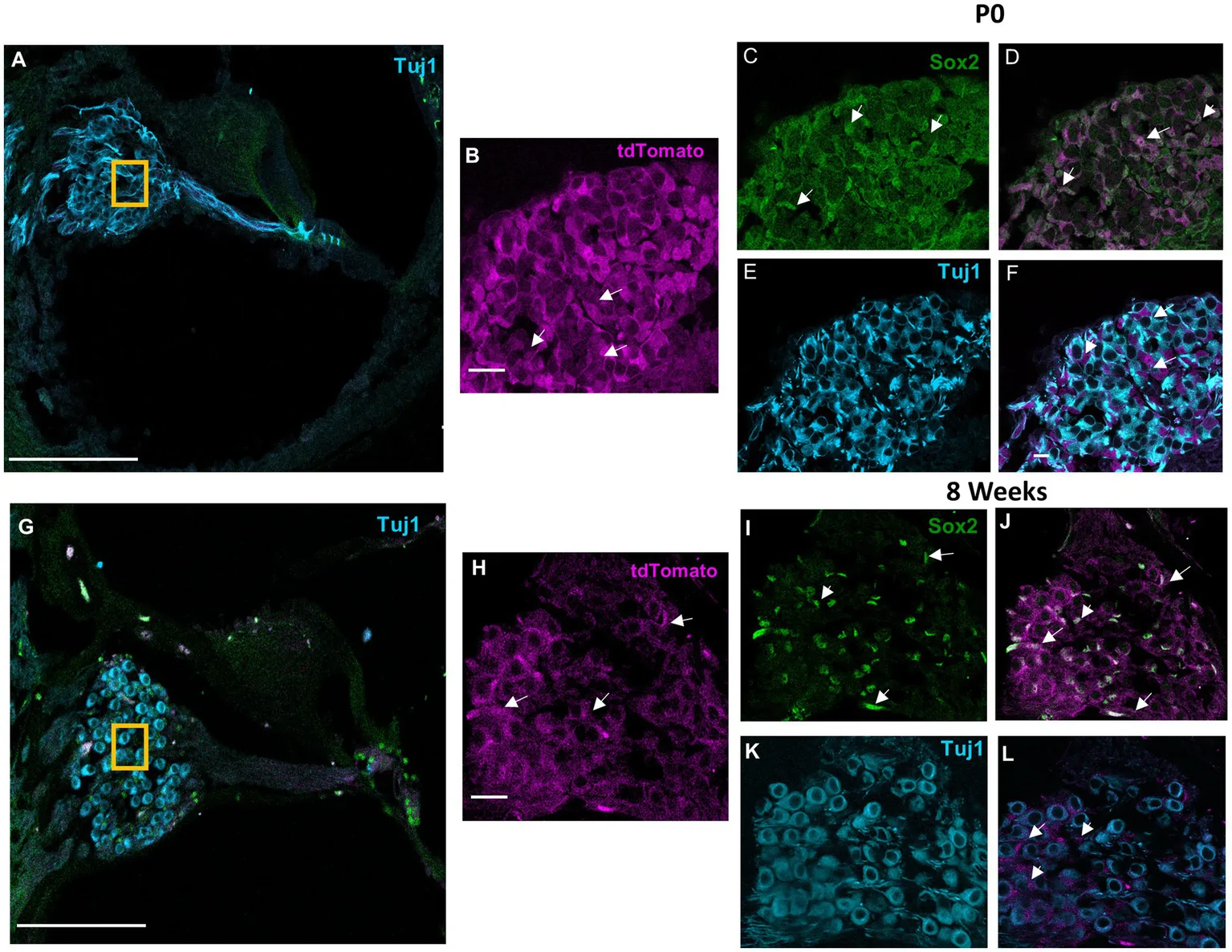
AhR is expressed in the satellite glia surrounding spiral ganglion neurons at neonatal and adult ages. Confocal images of mid-modiolar sections from the middle turn at P0 and 8 weeks of age. **(A)** SGN projections (labeled by Tuj1, blue) to the organ of Corti at P0. **(B–D)** tdTomato (magenta, arrows) is expressed in the satellite glia labeled by Sox2 (green) at P0 (*n* = 6). **(B–F)** tdTomato is not expressed in the SGNs labeled by Tuj1 at P0. **(G)** SGN projections to the organ of Corti at 8 weeks of age (*n* = 6). **(H–J)** tdTomato (arrows) is expressed in the satellite glia labeled by Sox2 at 8 weeks of age. **(H–L)** tdTomato is not expressed in the SGNs labeled by Tuj1 at 8 weeks of age. Scale bar: 200 μm in **(A)**, **(G)**; 20 μm in **(B–F)** and **(H–L)**.

### 3.2 AhR is upregulated after neomycin exposure

In one RNAseq study using the chicken basilar papilla (which is similar to the mouse cochlea), *AhR* expression was increased in response to neomycin-induced hair cell damage (Hawkins et al., 2007) suggesting that AhR might have a role in the cellular processes that occur after hair cell injury and during regeneration. Therefore, we hypothesized that the expression of AhR would increase in the organ of Corti post-damage. To test this, we used cochlear explants created from *Ahr^tdTomato^* pups at P2. Explants were equilibrated overnight followed by treatment with 600 μM neomycin for 24 h (Figure 3A). The tdTomato signal was very low in the explants treated with media only (*n* = 8), however 1 out of 8 media treated controls showed a moderate number of tdTomato-positive cells in the basal region which may have been caused by mechanical injury to the hair cells during the microdissection of the cochlea. We observed increased expression of tdTomato in all the cochlear explants (9 out of 9) treated with neomycin for 24 h compared to the explants treated with media only (Figures 3B–J’). The tdTomato expression was present in a subset of Sox2-positive supporting cells located in the regions lateral and medial to the hair cells in the explants treated with neomycin (Figures 3E–J’). Note that tdTomato is expressed in the entire cell, while Sox2 only labels the nuclei of supporting cells.

**Figure 3:**
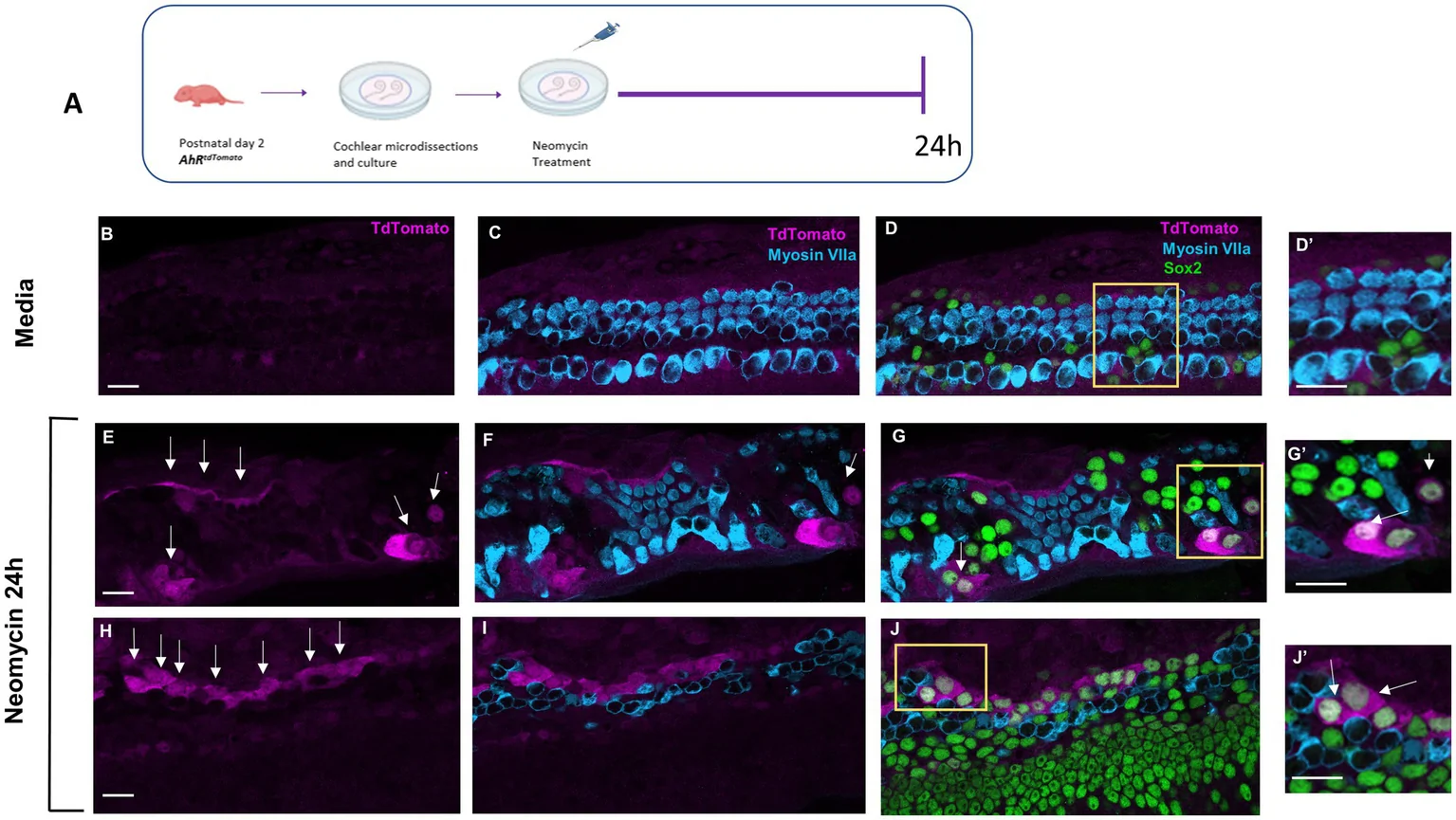
Expression of AhR is increased after exposure to neomycin. **(A)** Schematic of the explant culture and neomycin treatment. Image created using BioRender. **(B–J’)** Confocal images showing the expression of tdTomato (magenta), Sox2-positive (green) supporting cells and myosin VIIa-positive (blue) hair cells in media treated controls (*n* = 8) (**B**–**D′)** and neomycin treated explants (*n* = 9) (**E**–**J’)**. Explants treated with neomycin show increased expression of tdTomato (arrows) in some of the supporting cells in the regions medial **(E**–**G’)** and lateral **(H**–**J’)** to the hair cells. Scale bar: 20 μm in **(B–D)**, **(E–G)**, and **(H–J)**; 5 μm in **(D′, G’, J’)**.

To determine which supporting cell subtypes had increased tdTomato expression after neomycin treatment, we performed immunostaining to label Hensen cells with brain lipid binding protein (BLBP), also called fatty acid binding protein 7 (Saino-Saito et al., 2010; Chrysostomou et al., 2020), and the cytoplasm of pillar and Deiters’ cells using S100A1 (Coppens et al., 2001; White et al., 2006; McGovern et al., 2018). We observed co-labeling of tdTomato with S100A1 in the second and third row of Deiters’ cells in all explants. (Figures 4A–E’). In the Hensen cell region, the BLBP-positive cells were in close proximity with tdTomato-positive cells (Figures 4F–J’), with some explants (2 out of 7) showed co-labeling of BLBP with tdTomato in some cells. We also used real-time qPCR to confirm our results with the *Ahr^tdTomato^* reporter line and found a significant increase in *AhR* expression in the explants treated with neomycin for 24 h compared to explants treated with media only (Figure 4K).

**Figure 4:**
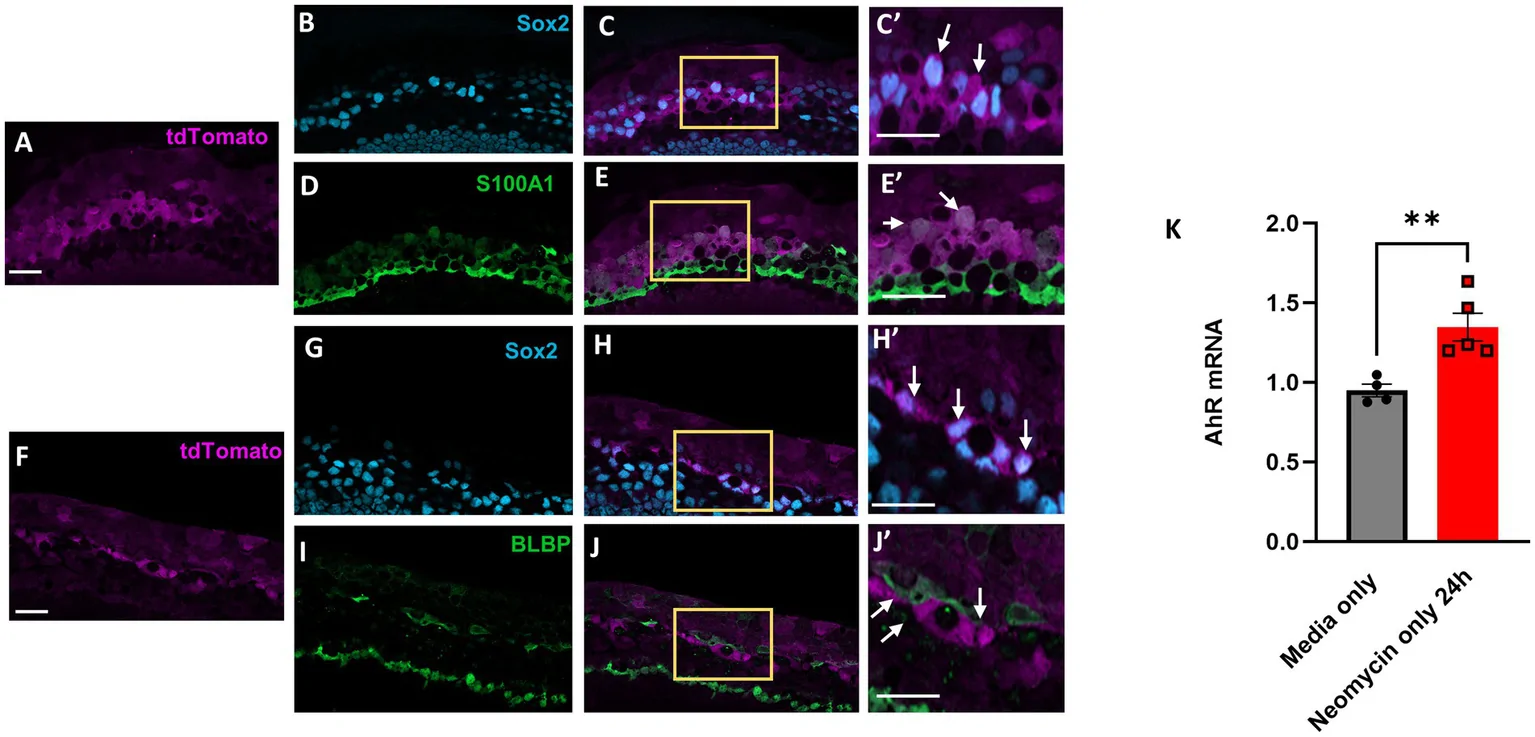
Expression of AhR is increased in Deiters’ cells after neomycin treatment. Confocal images showing the expression of tdTomato (magenta), Sox2-positive (blue) supporting cells, S100A1-positive Deiters’ cells (green in **D–E’**) and BLBP-positive Hensen cells (green in **I–J’**). Neomycin treated explants (*n* = 7) show increased expression of tdTomato (arrows) in Sox2-positive supporting cells **(A–C′, F–H′)**. Co-labeling of S100A1 with tdTomato was observed in the second and third row of Deiters’ cell labeled by S100A1 (**A, D**–**E’**). tdTomato was observed in close proximity to Hensen’s cells **(F, I–J’)**. **(K)** Expression level of *AhR* mRNA was increased in the groups treated with neomycin for 24 h compared to media only controls (*N* = 4–5). *p = 0.0068 as measured by an unpaired Student’s *t* test. Scale bar: 20 μm in **(A–C, D–F, G–I, and J–L)**; 5 μm in **(C′, F′, I′, L’)**.

### 3.3 ATP is increased after treatment with neomycin

Although neomycin uptake occurs primarily in hair cells (Alharazneh et al., 2011; Kitcher et al., 2019; Kim and Ricci, 2022), we observed increased tdTomato expression in neighboring supporting cells, including Hensen and Deiters’ cells. These findings suggest that injured hair cells transmit signals to supporting cells during ototoxin-induced injury. Previous studies have shown that adenosine 5′-triphosphate (ATP) is released from hair cells in response to mechanical injury or neomycin treatment (Lahne and Gale, 2008a, 2010; Cheng et al., 2023). We measured the concentration of ATP in cochlear explants treated with neomycin or media for 24 h using a luciferase-based ATP assay (Figure 5A). In control samples treated with media only (*n* = 4), ATP levels were low, however, the ATP concentration increased 3-fold in explants treated with neomycin (Figure 5B) (*n* = 4). These results suggest that neomycin induces significant ATP levels that could be released from hair cells during injury.

**Figure 5:**
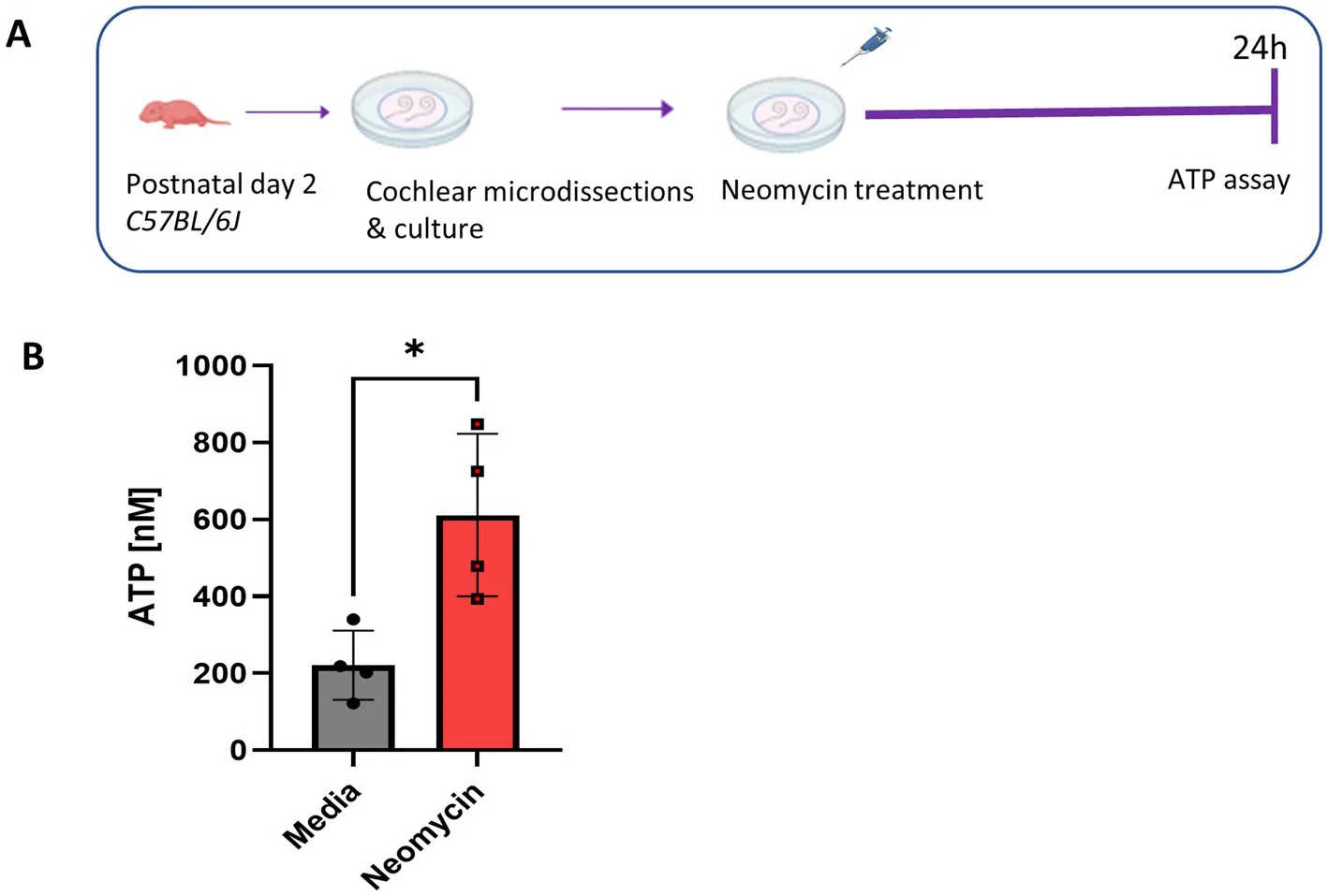
ATP levels are increased after neomycin treatment. **(A)** Schematic of the explant and neomycin treatment used for the ATP assay. Image created using BioRender. **(B)** ATP concentration was significantly increased in cochlear explants treated with neomycin (*n* = 4) compared to media treated controls (*n* = 4). *p = 0.0145 as measured by an unpaired Student’s *t* test.

### 3.4 AhR expression is increased after treatment with ATP

To further understand if ATP mediates AhR upregulation, we treated explants with 100 μM ATP for 24 h (Figure 6A) and observed an increase in tdTomato expression in Sox2-positive supporting cells (Figures 6B–N’). The tdTomato expression was colocalized with S100A1 in all the explants (6 out of 6) treated with ATP, specifically in the third row of Deiters’ cells (Figures 6H–I’). Immunostaining with BLBP also showed co-labeling with tdTomato suggesting expression of AhR in Hensen cells (Figures 6J–N’). The tdTomato signal was very low in the explants treated with media only (Figures 6B–D’), however 1 out of 6 media treated controls showed a moderate number of tdTomato-positive cells in the basal region. To confirm the requirement of purinergic signaling for AhR upregulation, we co-administered neomycin with the P2 receptor antagonist suramin (1 mM) to *AhR^tdTomato^* cochlear explants for 24 h (Figure 7A). No tdTomato expression was seen when suramin was present (Figures 7F–H), suggesting that blockade of purinergic receptors eliminated the damage-induced expression of AhR in supporting cells. Since damaged hair cells release ATP and the AhR expression pattern after treatment with both ATP and neomycin were similar, our findings support a model in which ATP mediates communication between injured hair cells and supporting cells by AhR signaling. (Figure 7I).

**Figure 6:**
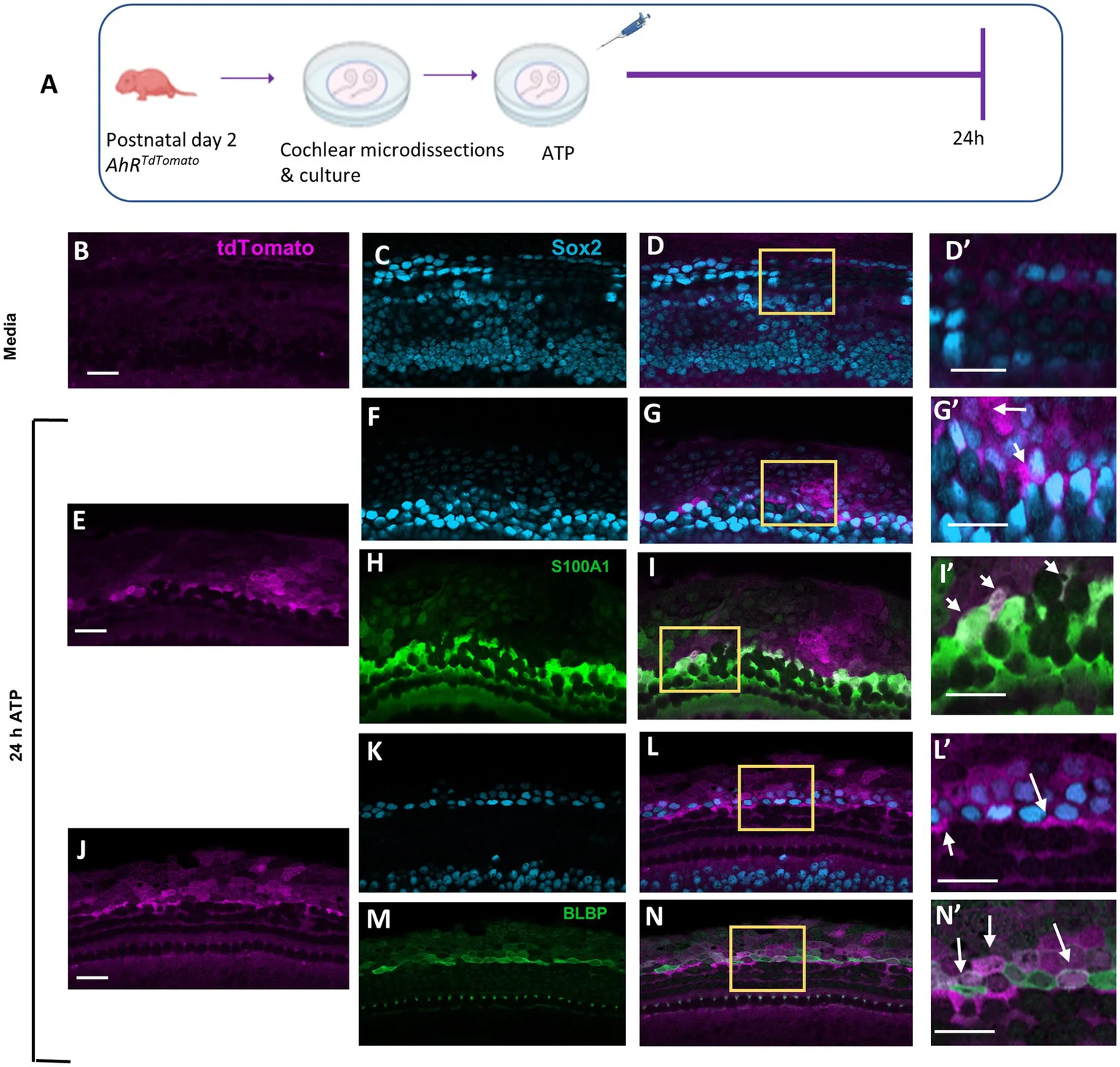
Expression of AhR is increased in Deiters’ and Hensen cells after ATP treatment. **(A)** Schematic of the explant culture and neomycin treatment. Image created using BioRender. **(B–N′)** Confocal images showing the expression of tdTomato (magenta), Sox2-positive (blue) supporting cells, S100A1-positive Deiters’ cells (green in **H–I′**) and BLBP-positive Hensen cells (green in **M–N′**). There was no tdTomato expression in media treated controls (*n* = 6) (**B**–**D′).** ATP-treated explants had increased expression of tdTomato in some Sox2-positive supporting cells **(E**–**G′, J**–**L′)**. Co-labeling of S100A1 with tdTomato (arrows in **I′**) was observed in third row of Deiters’ cell labeled by S100A1 **(E, H**–**I′)**. Co-labeling of BLBP with tdTomato (arrows in **N′**) was also observed in Hensen cells **(J, M**–**N′)**. Scale bar: 20 μm in **(B–N)**; 5 μm in **(D′, G’, I’, L’, N′)**.

**Figure 7:**
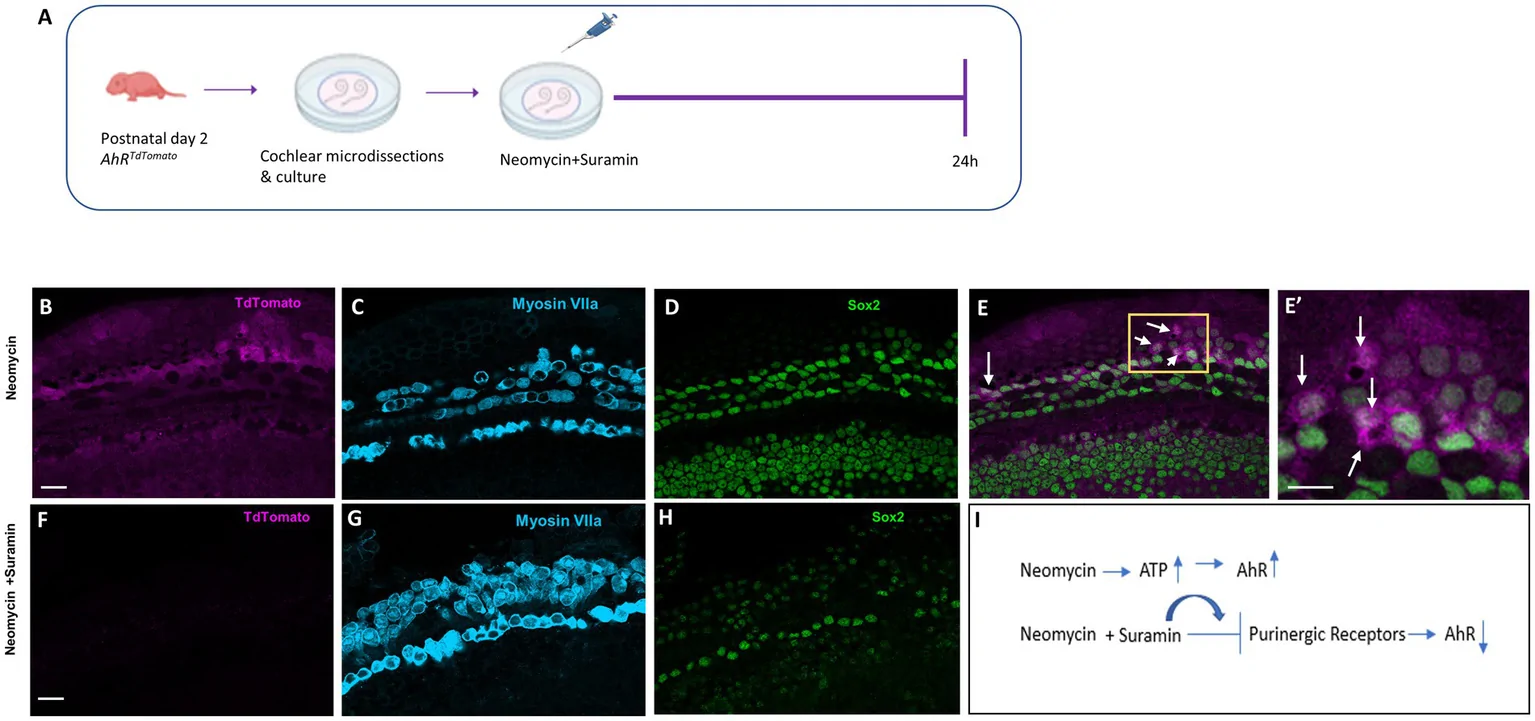
Upregulation of AhR is eliminated after co-incubation of suramin with neomycin. **(A)** Schematic of the explant culture and treatment with neomycin and suramin. Image created using BioRender. **(B–H)** Confocal images showing the expression of tdTomato (magenta), Sox2-positive (green) supporting cells and myosin VIIa-positive (blue) hair cells in explants. Neomycin treatment increased tdTomato expression in supporting cells lateral to the hair cells (*n* = 6) (**B–E′**, arrows). The co-incubation of neomycin and suramin (purinergic receptor antagonist) prevented changes in tdTomato expression (*n* = 6) **(F–H)**. **(I)** Schematic of the pathway from neomycin treatment to AhR upregulation. Scale bar: 20 μm in **B–H**; 5 μm in **E′**.

### 3.5 AhR expression is increased after noise exposure

Emerging evidence has identified anti-oxidative functions of AhR in different organ systems (Dietrich, 2016; Edamitsu et al., 2022). Given that acoustic trauma significantly elevates reactive oxygen species (ROS) levels, (Van Campen et al., 2002; Yamashita et al., 2004; Kurabi et al., 2017; Kühl et al., 2019), we examined the spatiotemporal changes in AhR expression following noise exposure. *AhR^tdTomato^* mice were exposed to noise (105 ± 5 dB SPL for 1 h) at 8 weeks of age and examined at 1 h and 24 h post-noise (*n* = 8). There were no tdTomato-positive cells in the organ of Corti in unexposed controls (Figures 8A–D’). At 1 h after the noise exposure, we observed an increase in tdTomato expression and loss of some outer hair cells (Figures 8E–H’). Unlike explants treated with neomycin, most Sox2-positive supporting cells exhibited tdTomato expression in the noise-exposed cochlea. While hair cell damage was not compared between models, this difference likely results from the distinct experimental conditions. *In vivo* acoustic trauma induces severe, widespread mechanical and inflammatory stress that drives circulating immune cell infiltration (Hirose et al., 2005; Tornabene et al., 2006; Shin et al., 2022). Conversely, *in vitro* neomycin treated explants represent a localized chemical insult completely isolated from systemic immune responses, which may limit the secondary cellular stress signals.

**Figure 8:**
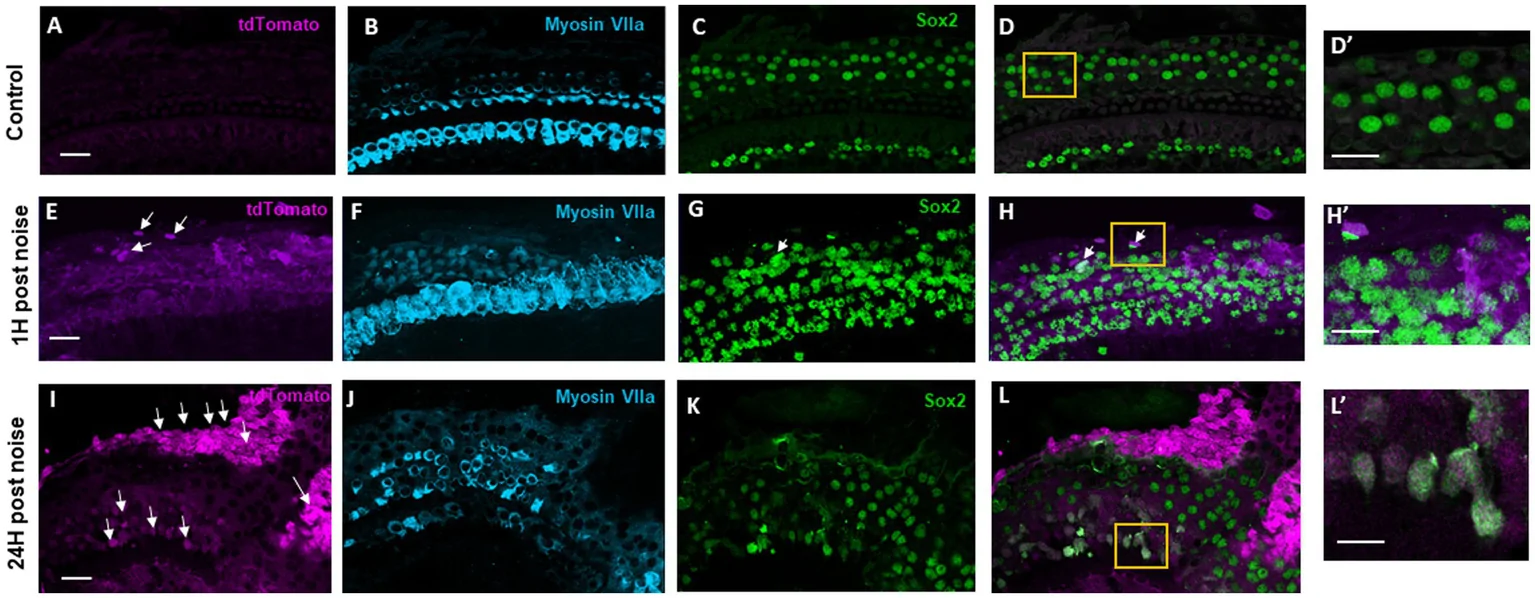
Expression of AhR is increased after noise exposure. Confocal images showing the expression of tdTomato (magenta), Sox2-positive (green) supporting cells and myosin VIIa-positive (blue) hair cells in cochlea from mice at 8 weeks of age. **(A–D)** The expression of tdTomato was very low in the organ of Corti of controls that were not exposed to noise (*n* = 8). **(E–H′)** TdTomato expression was increased at 1 h after noise exposure (*n* = 8) in the majority of Sox2-positive supporting cells (arrows) in the basal turn. **(I–L′)** TdTomato expression was increased 24 h after noise exposure in some Sox2-positive supporting cells and in the outer sulcus region (arrows). Scale bar: 20 μm in **A–L**; 5 μm in **D′, H′, and L′**.

At 24 h post-noise exposure, a large number of tdTomato-positive cells were observed in the outer sulcus, lateral to outer hair cells, while tdTomato expression in supporting cells within the organ of Corti was reduced compared to the 1-h timepoint (Figures 8I–L’). Because the cochleae were harvested at acute timepoints (1 and 24 h post-exposure) and previous studies have shown that it takes several days to weeks for hair cells to degenerate and die (Bohne and Harding, 2000; Yamashita et al., 2004; Kujawa and Liberman, 2009; Gratias et al., 2021), we did not quantify the extent of hair cell damage in our samples. However based on previous reports, the noise exposure paradigm that we used should kill less than 5% of outer hair cells (Blum et al., 2024). Notably, the noise-induced changes and the tdTomato expression pattern were observed in all cochlear turns.

### 3.6 Endogenous *AhR* activity regulates hair cell survival after neomycin exposure

To determine the function of endogenous AhR in the context of neomycin exposure, we deleted *AhR* from the organ of Corti using *Sox2^CreER^:AhR^loxP/loxP^* mice that received tamoxifen (3 mg/40 g, IP) at P0. With this age of tamoxifen induction *Sox2^CreER^* is expressed in all supporting cells and the majority of hair cells (Walters et al., 2015; McGovern et al., 2017). First, we confirmed deletion of *AhR* from the organ of Corti in *Sox2^CreER^:AhR^loxP/loxP^* mice. We observed a ~ 50% reduction in *AhR* mRNA levels compared to Cre-negative controls (Figure 9B). This level of reduction is consistent with the CreER line targeting Sox2-positive cells and the presence of Sox2-negative cells in the cochlea. Next, explant cultures were created from *Sox2^CreER^: AhR^loxP/loxP^* mice and Cre-negative controls at P2 and treated with neomycin or media for 24 h, followed by media washes and further incubation for 24 or 48 h (Figure 9A). We quantified total hair cell numbers and compared numbers between groups. At 24 h post-neomycin exposure, there were no differences in the number of hair cells between the media treated controls (*n* = 4–5) and the Cre-negative explants treated with neomycin (*n* = 7–8). However, we observed a significant amount of hair cell loss in the basal turn of the explants with *AhR* deletion compared to both media treated controls and neomycin treated controls with normal *AhR* expression (Cre-negative mice) (Figure 9C). At 48 h post-neomycin exposure, we observed a significant loss of hair cells in the middle and basal turns in the neomycin treated Cre-negative explants with normal *AhR* expression compared to media treated controls. For *Sox2^CreER^: AhR^loxP/loxP^* explants, there was a significant decrease in hair cell numbers in the middle turn compared to media treated controls. In the basal turn, we found a larger amount of hair cell loss after neomycin treatment in the explants with *AhR* deletion compared to both media treated controls and neomycin treated explants with normal *AhR* expression (Cre-negative mice) (Figure 9D).

**Figure 9:**
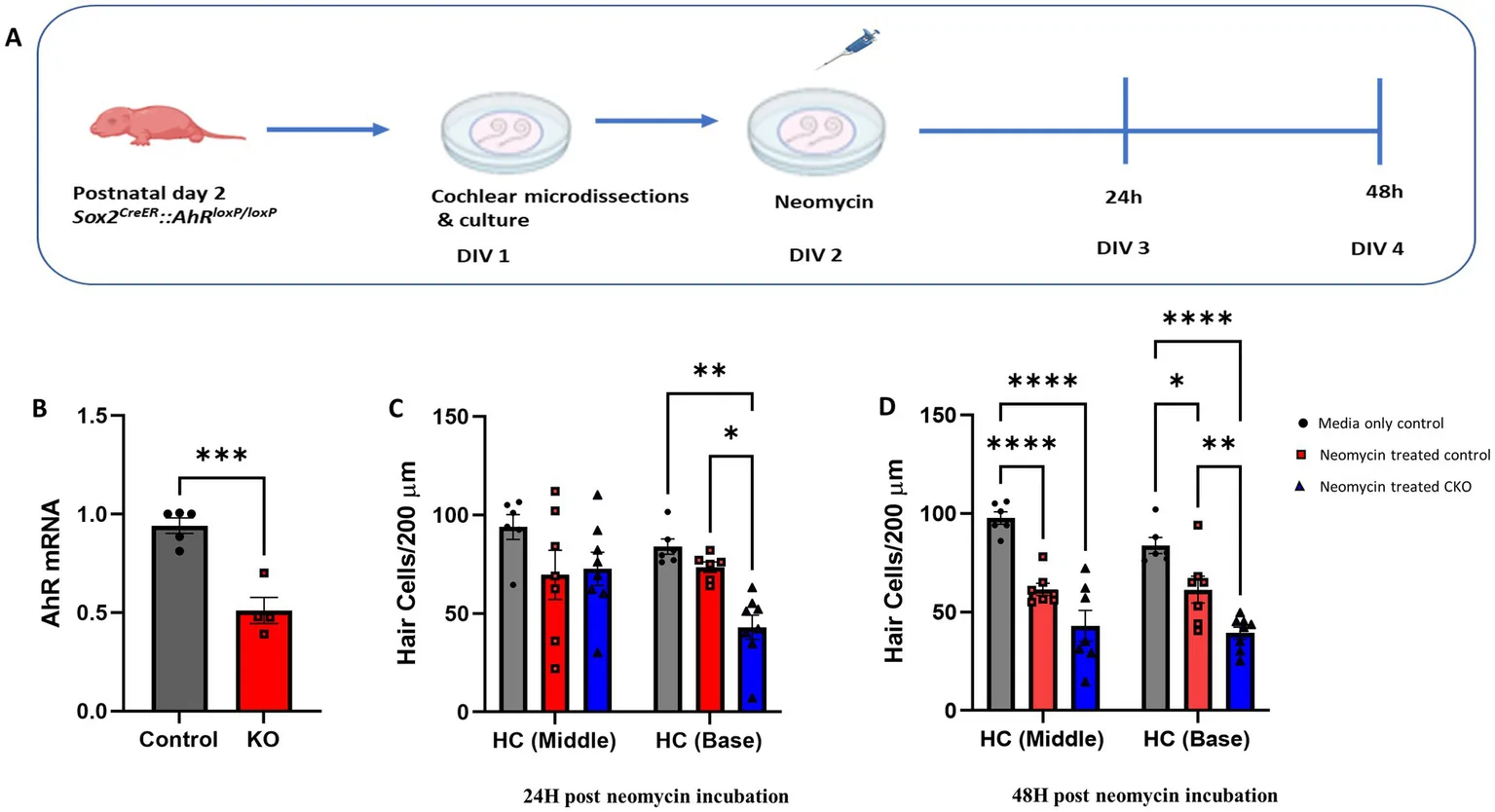
Deletion of AhR increases hair cell loss upon neomycin exposure. **(A)** Schematic of the explant culture and neomycin treatment. Image created using BioRender. **(B)** Expression level of *AhR* mRNA was reduced by ~50% in *Sox2^CreERT2^:AhR^loxP/loxP^* mice compared to Cre-negative controls (*n* = 4–5). ***p = 0.0006 determined by an unpaired Student’s *t* test. **(C)** Significant hair cell loss was observed in the basal turn of explants from *Sox2^CreERT2^: AhR^loxP/loxP^* mice treated with neomycin (*n* = 7–8) compared to Cre-negative controls treated with neomycin (*n* = 7) and media treated controls (*n* = 6) at 24 h post-neomycin treatment. *p = 0.0232, **p = 0.0019. **(D)** At 48 h post neomycin treatment, significant hair cell loss was observed in the middle and basal turns of Cre-negative control explants treated with neomycin (*n* = 7) compared to media treated controls (*n* = 6). *p = 0.0351, ****p < 0.0001. In the basal turn, hair cell loss was increased further in *Sox2^CreERT2^:AhR^loxP/loxP^* mice treated with neomycin (*n* = 7–8) compared to neomycin treated Cre-negative controls (*n* = 7). *p = 0.0129, **p = 0.0084, ****p < 0.0001, based on a two-way ANOVA followed by a Tukey’s multiple comparison test. A significant main effect of genotype was observed at 24 h after neomycin treatment [*F* (2, 35) = 8.063, *p* = 0.0013] and at 48 h after neomycin treatment [*F* (2, 35) = 45.95, *p* < 0.0001].

## 4 Discussion

Here we provide the first report of the expression pattern of AhR in the cochlea. Under normal conditions, AhR expression was very low in the organ of Corti in all cochlear turns at the postnatal ages we examined, however, robust expression was observed in Schwann cells and satellite glial cells surrounding spiral ganglion neurons at P0. At 8 weeks of age, AhR expression became a mosaic pattern in these glia. We also found tdTomato-positive cells beneath the inner hair cell layer, medial to inner border and inner phalangeal cells at both ages. This expression pattern, location and ovoid cellular shape aligns closely with the “unique cell population” identified as nestin-positive cells (Chow et al., 2015; Smeti et al., 2011). In support, nestin expression has been shown to co-localize with AhR expression in granule neurons (De La Parra et al., 2018) and dorsal root ganglion neurons (Halawani et al., 2026).

Multiple RNAseq studies from the neonatal cochlea show that *AhR* is expressed in non-sensory supporting cells such as Dieters’ cells, Hensen cells, inner pillar cells and inner phalangeal cells under normal conditions (Cai et al., 2015; Kolla et al., 2020). Our observation of low AhR expression measured by the *AhR^tdTomato^* reporter mouse line may suggest differences between RNA and protein expression, since there are many posttranslational mechanisms that regulate protein levels (Walters and Cox, 2019). The AhR localization in the cochlea we observed aligns with the established presence of AhR in the CNS and other sensory tissues. Specifically, AhR expression is present in glial populations such as astrocytes (Filbrandt et al., 2004b; Zhou et al., 2021; Sha et al., 2025) and microglia (Zhou et al., 2021; Zähringer et al., 2025). Furthermore, previous reports have demonstrated that AhR regulates myelin gene expression and AhR’s deficiency leads to thinner myelination in the experimental autoimmune encephalomyelitis preclinical model of multiple sclerosis (Shackleford et al., 2018; Rothhammer et al., 2021b).

In the cochlear explants treated with neomycin, AhR expression was increased in a subset of supporting cells specifically in the region medial and lateral to hair cells after 24 h of exposure compared to media treated controls. AhR is traditionally recognized as a transcription factor that senses xenobiotics and environmental toxins (Köhle and Bock, 2007; Bortoli et al., 2018; Zhang et al., 2019), however, its upregulation in these supporting cells within 24 h of neomycin exposure suggests that AhR acts as a sensor of hair cell injury. Our findings are similar to reports where AhR-positive non-neuronal cells were observed as early as 18 h after traumatic brain injury (Xu et al., 2016). Another study reported that AhR activation was induced during neuronal stress, showing a significant increase in phosphorylated AhR-positive astrocytes and microglia (Zhou et al., 2021). The physiological functions of AhR in the CNS is demonstrated by studies where disrupting expression of *AhR* decreased cell survival and neuronal differentiation (Latchney et al., 2013b). In injury and neuroinflammatory contexts, astrocytic AhR limits excessive NF-κB-driven signaling thereby reducing inflammation and contributing to neuronal survival (Vogel et al., 2014; Lee et al., 2015; Ojo and Tischkau, 2021, 2025; Guo et al., 2024; Sun et al., 2026b).

It is well-established that hair cell damage causes a rise in intracellular calcium levels in supporting cells mediated by extracellular ATP (Piazza et al., 2007; Lahne and Gale, 2008a, 2010; Ye et al., 2016; Wood et al., 2024). Supporting cell subtypes such as Deiters’ and Hensen cells express metabotropic purinergic receptors such as *P*2*Y*2 (Piazza et al., 2007; Vlajkovic and Thorne, 2022). Extracellular application of ATP to the endolymphatic surface of Hensen cells in the isolated cochlea showed the co-activation of both ionotropic and metabotropic receptors. The first and faster inward current was mediated by direct cation influx through ionotropic P2X receptors and the second, slower current was from metabotropic P2Y receptor activation, which triggers an IP3-mediated release of calcium from intracellular stores (Lagostena et al., 2001). In another study, during damage to cochlear explants, slow calcium waves propagated between Deiters’ cells, outer sulcus cells, and Kolliker’s organ and faster waves were observed in a subset of cells in the hair cell region which were mediated by ATP (Lahne and Gale, 2010). Also, in response to neomycin, the purinergic receptor, P2X7R, was upregulated in supporting cells (Cheng et al., 2023). ATP-mediated calcium influx is required for other damage-induced pathways in the cochlea, such as the activation of ERK1/2 in Deiters’ and inner phalangeal cells, which serves to promote wound repair and regulates hair cell survival (Lahne and Gale, 2008b). Similarly, in response to stretch-induced injury in primary cultures of the cortical astrocytes, ERK activation was observed (Neary et al., 2003).

We hypothesize that the mobilization of intracellular calcium in supporting cells, triggered by extracellular ATP released by injured hair cells, acts as the upstream signaling event that couples hair cell injury to the upregulation of AhR in supporting cells (Figure 10). This hypothesis is supported by research across multiple biological systems where increased calcium signaling leads to activation of transcription factors and regulation of gene expression. In neurons, calcium-activated signaling pathways mediate transcription of genes such as bone-derived neurotrophic factor (BDNF) (West et al., 2001). Calcium signals are also known to induce “immediate-early” response genes like *c-fos* (Cruz-Mendoza et al., 2022). Also, after calcium entry into the cytosol, activation of NF-κB in primary cultures of neonatal cerebellar granule neurons is observed as a downstream sequence (Lilienbaum and Israel, 2003). Studies report that NF-κB directly binds to the AhR promoter to increase *AhR* mRNA levels in intestinal Caco-2 cells (Champion et al., 2013) suggesting a mechanistic link for how increased calcium signaling acts through NF-κB to upregulate AhR expression. However, this process has not been tested in the cochlea.

**Figure 10:**
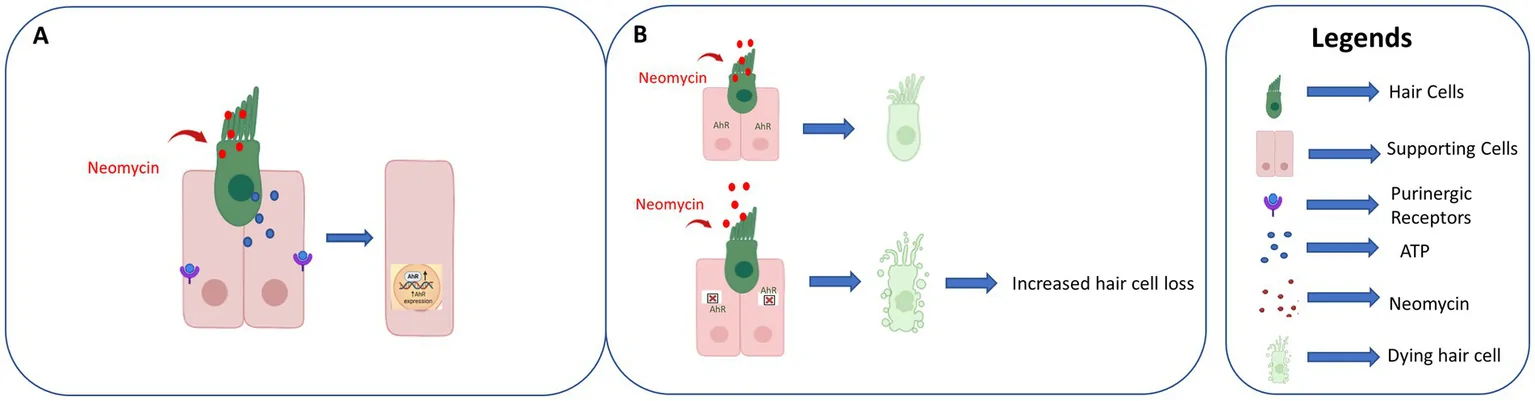
Schematic of AhR signaling in the cochlea after neomycin exposure. **(A)** Neomycin enters hair cells and triggers an increase in ATP levels which causes release of ATP. Released ATP binds to purinergic receptors on supporting cells leading to increased expression of AhR in supporting cells. **(B)** Treatment with neomycin in cochlear explants with *AhR* deletion results in increased hair cell loss compared to control explants with normal *AhR* expression. Image created using BioRender.

The coordinated response of supporting cells to hair cell injury, as evidenced by increased AhR expression in Hensen and Deiters’ cells in our study likely represents a homeostatic or protective response. Our observation of increased hair cell loss upon neomycin treatment in the explants with *AhR* deletion from the organ of Corti suggests that AhR activation by endogenous ligands reduces neomycin-induced hair cell death. This paracrine signaling we observed is similar to the cochlea’s innate antiviral defense mechanism, where supporting cells and greater epithelial ridge (GER) cells sense viral infection and produce interferon-α/β (IFN-α/β) to activate survival-regulating receptors (*Ifnar1/Ifnar2*) on the hair cells (Hayashi et al., 2020). In addition, cultured heat-shocked utricles showed protective effects against gentamicin-induced hair cell death compared to non-heat-shocked controls (May et al., 2013). Further investigation into the underlying mechanisms revealed that heat exposure caused supporting cells to upregulate heat shock protein (HSP) 70 that was released in exosomes, and taken up by hair cells through paracrine signaling (Breglio et al., 2020). These findings highlight the role of non-sensory cells acting as homeostatic regulators of injury to mediate the survival of hair cells during stress.

The observation of increased AhR expression in the cochlea at 1 and 24 h following noise exposure suggests that AhR is an early responder in the molecular pathogenesis of noise-induced hearing loss (NIHL). This upregulation is also similar to the early elevation of ROS that occurs upon acoustic trauma (Van Campen et al., 2002; Kurabi et al., 2017; Kühl et al., 2019). Given that ROS accumulation is a primary driver of hair cell apoptosis and permanent hearing loss, we hypothesize that increased AhR expression could be a response from supporting cells to decrease early oxidative damage. Further research into AhR’s function in the cochlea could provide novel insights into otoprotective mechanisms and potential therapeutic targets for hearing loss.

In the CNS, *AhR* deficiency enhanced astrocytes’ sensitivity in primary astrocyte culture to LPS-induced inflammatory stimuli (Ojo and Tischkau, 2025) suggesting a role of AhR in astrocytes in limiting neuroinflammation. Given that astrocytes and supporting cells share multiple roles including stress response by ATP-mediated calcium signaling, inflammatory response and homeostasis (Charles et al., 1991; Salter and Hicks, 1994; Gallagher and Salter, 2003; Ceriani et al., 2016; Pang et al., 2022; Cheng et al., 2023; Wood et al., 2024; Han et al., 2025; Ye and Quintana, 2026), it is possible that deletion of *AhR* from the organ of Corti increased the sensitivity to neomycin-induced hair cell loss as observed in our study (Figure 10). Furthermore, *AhR* deficient cells are inherently susceptible to inflammation even in the absence of strong stimuli (Ojo and Tischkau, 2025). This suggests that exposure to neomycin in the absence of *AhR* further increased inflammation and oxidative stress leading to the increased hair cell loss as observed in our experiment.

Overall, this study highlights the expression pattern of AhR at neonatal and adult ages and the dynamic response of AhR after neomycin treatment and noise exposure. Furthermore, this study identifies a unique response of supporting cells by upregulating AhR during hair cell injury that is mediated by ATP signaling. The increased hair cell loss in *AhR* deficient cochlea suggests endogenous role of AhR to regulate hair cell survival.

## Data Availability

The original contributions presented in the study are included in the article/supplementary material, further inquiries can be directed to the corresponding author.
